# Sebaceous gland tumors and internal malignancy in the context of Muir-Torre syndrome. A case report and review of the literature

**DOI:** 10.1186/1477-7819-4-8

**Published:** 2006-02-08

**Authors:** K Tsalis, K Blouhos, K Vasiliadis, T Tsachalis, S Angelopoulos, D Betsis

**Affiliations:** 1 Fourth Department of Surgery, Aristotle University of Thessaloniki, Greece

## Abstract

**Background:**

The Muir-Torre syndrome is a rare autosomal dominant condition and is currently considered a subtype of the more common hereditary nonpolyposis colorectal cancer syndrome, in which multiple primary malignancies occur together with sebaceous gland tumors.

**Case presentation:**

We describe a case of a 62-year-old woman with three primary colorectal tumors, genital tumor, and sebaceous adenomas and present her family history of three generations. Our case represents the first case reported from Greece in the international literature.

**Conclusion:**

Recognition of the syndrome in patients with sebaceous gland tumors should facilitate early detection of subsequent malignancies if the patient is entered into appropriate screening programs.

## Background

Distinguishing cutaneous signs, which are associated with hereditary cancer syndromes, are known as cancer-associated genodermatoses. The Muir-Torre syndrome (MTS) is an example, and is defined by the combination of at least one sebaceous adenoma, epithelioma or carcinoma and at least one visceral carcinoma occurring in the same individual in the absence of other precipitating factors such as radiotherapy or AIDS [1]. Individuals with the syndrome may develop multiple primary malignancies at different sites. Diagnosis of MTS has implications for cancer screening and surveillance in the affected individual and his or her relatives [2,3]. Our case represents the first case reported from Greece in the international literature.

## Case presentation

A 62-year-old woman with a past history of adenocarcinoma of the rectum and genital cancer, underwent abdominoperineal resection with a permanent left ostomy and total hysterectomy in the age of 37. Some 9 years later she was found to have colonic polyps with malignant transformation and was treated by surgical resection (colotomy and polypectomy).

In 1993 and 1997 she was found to have benign colonic polyps which were excised endoscopically, and in March and June 2003 she was referred to our surgical outpatient department with subcutaneus lesions, first on the left side of the abdominal wall and then on the right buttock. The lesions were excised under local anesthesia and were reported to be sebaceous adenomas, and the diagnosis of the Muir-Torre syndrome (MTS) was suggested. Ever since the patient was commenced on surveillance program that included annual clinical examination, CEA evaluation, chest radiography, urine cytology, colonoscopy and mammography.

In June 2005, an adenocarcinoma of the ascending colon was detected. An abdominal computed tomography (CT) scan showed no evidence of metastatic disease or paraaortic lymphadenopathy. A right hemicolectomy was performed, and the pathology report confirmed a poorly differentiated mucinous adenocarcinoma (stage II, T_3_N_0_M_0_, Dukes B). Screening tests for further malignancies, including chest radiography and upper GI endoscopy were all negative, and none of the tumor markers were elevated.

The patient has twin sons both of whom are in their early forties and currently healthy. She had three siblings from that only one brother is alive without cancerous history. One brother underwent a subtotal gastrectomy in his forties for gastric cancer and died in his fifties of a metachronous primary gastric cancer in the oesophagogastric junction. Another brother is alive, who has undergone a colon resection twice, first in his twenties and then 22 years later for colon cancer, and has a history of multiple sebaceous adenomas. Her mother had undergone a colon resection in her forties for colon cancer and developed at least seven primary cancers of colon, genitourinary, upper GI tract, sebaceous adenomas-epitheliomas, during a 21-year period. She died in her sixties of a second gastric cancer. Her grandfather died in his forties from colon cancer. Further family history is less clear, but the patient believes that some other members of her family died of cancers at various sites. Ages at diagnosis of cancer ranged from 29 to 62 years (median age: 48 years).

**Figure 1:**
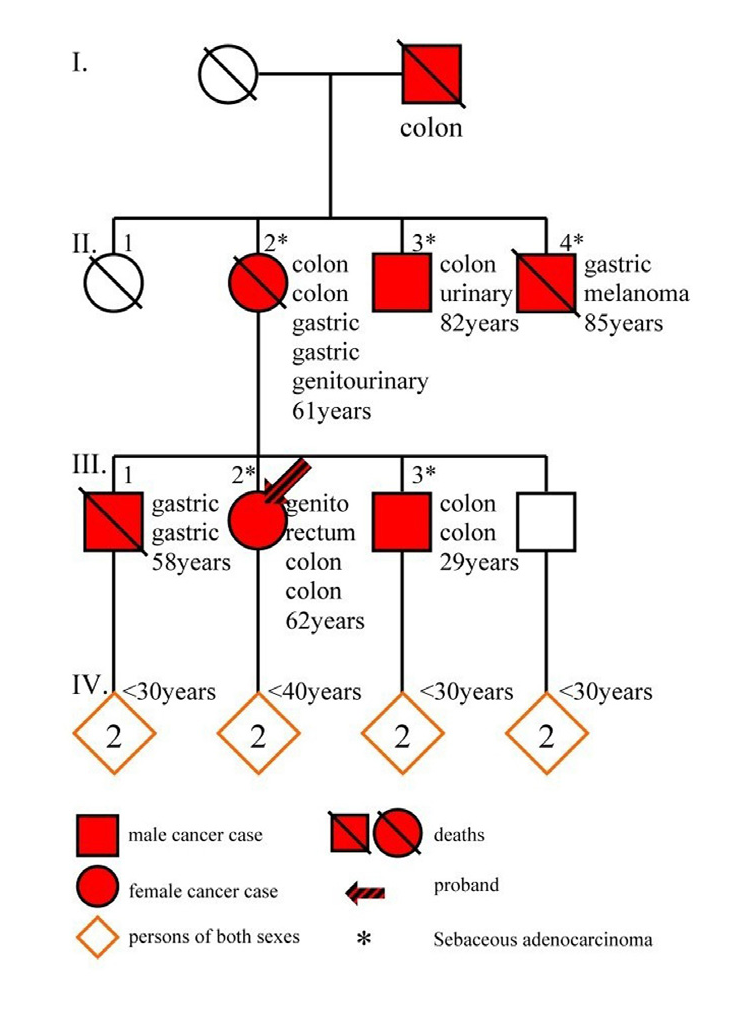
Condensed pedigree of four generations (I to IV) of the family. Patients II-2 and III-2 had four and three primary gastric and colorectal carcinomas respectively, as well as other forms of cancer. *Squares: men, circles: women, diamonds: persons of either sex*

All family members were suggested to undergo endoscopy and dermatological surveillance. A pedigree of four family generations is shown in Figure 1.

## Discussion

The Muir-Torre syndrome was independently described by Muir in 1967 and Torre in 1968, and has since been recognized as a subtype of Lynch Type II hereditary nonpolyposis colon cancer (HNPCC) [4,5]. Akhtar *et al *in their review study identified a total of 205 reported cases in the world literature [6]. In the same study the authors reported that the defining feature of this syndrome is the combination of sebaceous gland tumors and at least one visceral cancer, usually gastrointestinal or genitourinary carcinomas.

Clinically the diagnosis of MTS is based on the presence of at least one sebaceous gland tumor associated with at least one primary visceral malignancy, as it happened in our case. Alternatively, diagnosis of the syndrome can be made if the patient has multiple keratoacanthomas with multiple internal malignancies and a family history of MTS [7]. Muir-Torre syndrome can also be diagnosed by genetic testing. A high proportion of tumors from MTS patients show microsatellite instability (MSI) due to germline mutations in either of two DNA mismatch repair (MMR) proteins [8].

Typical skin tumors associated with this syndrome include sebaceous adenomas, epitheliomas and carcinomas. Keratoacanthomas and basal cell carcinomas with sebaceous differentiation may also occur. All these sebaceous gland tumors are rare in the general population and the finding of such a tumor may represent a marker for MTS and should prompt a search for occult malignancy [9]. In addition, fifty-six per cent of skin lesions in MTS occur after diagnosis of the first malignancy, 6% occur concomitantly and 22% of skin lesions occur as the first malignancy of the syndrome [6]. The cutaneous lesions may occur as long as 25 years before or 37 years after the internal malignancy and multiple primary carcinomas at different sites are characteristic of MTS so that up to 9 visceral cancers in one individual have been reported [10].

Colorectal cancer is the commonest visceral neoplasm to occur in MTS, and the most frequent initial cancer [3,10]. In common with other forms of HNPCC, colorectal malignancies in MTS are usually proximal in location and tend to have a more indolent course than other forms of colorectal cancer [10]. Fifty-one per cent of MTS patients develop at least one colorectal cancer, and multiple colorectal cancers are common, genitourinary cancers occur in 24% of individuals, mainly transitional cell carcinomas. Carcinomas of the endometrium, ovary, breast, parotid, upper GI tract and larynx, and hematological malignancies are also associated with MTS. As in the Lynch II syndrome, cancers occur at a relatively young age. Colonic polyps are found in more than 25% of MTS patients, and are especially prevalent in patients with colorectal carcinoma [11].

The genetic disorder in MTS is an autosomal dominant inherited germline mutation in one of the DNA mismatch repair genes, MSH1 and MSH2 [8,12]. It is inherited with a high degree of penetrance and variable expression. The syndrome occurs in both sexes, with a male to female ratio of 3:2. Children of an MTS individual, therefore, have a 50% risk of inheriting the cancer predisposition. In families where the germline mutation can be identified, those individuals who have inherited the mutation should be offered regular screening examinations. In those who can be demonstrated not to have inherited the germline mutation, cancer surveillance is not necessary [13].

Screening for malignancy at all possible sites is impractical in MTS given the wide range of associated malignancies, and screening should probably concentrate on the colorectum, female genital tract and possibly urinary tract. In some families the occurrence of certain other tumors would be an indication for other screening modalities, for example upper GI endoscopy [2].

Cohen *et al *[10] suggested that a search for internal malignancy should be undertaken in the following: 1) a patient in whom an MTS-associated sebaceous gland tumor has been documented, 2) a patient in whom MTS has been diagnosed and 3) family members of an MTS patient. They also suggested surveillance program for patients with MTS or MTS-associated sebaceous gland tumors included annual clinical examination, CEA, cervical smear, chest radiography, and urine cytology, colonoscopy or barium enema every 3–5 years, and for female patients mammography 1–2 yearly to age 50 and annually thereafter, and endometrial biopsy every 3–5 years. Other authors have suggested that colonoscopy should be more frequent in view of the high frequency of colonic cancer and its proximal predominance, and advocate annual colonoscopy from the age of 25 years [3]. Besides, MTS screening may be laborious but is not universally performed, and a search for mutations in either MSH1 or MSH2 is expensive and time consuming. One attractive alternative is the analysis of mismatch repair (MMR) protein expression as a surrogate for assaying for the respective gene mutations [12,14].

The indolent course of cancers occurring as part of MTS is often commented on but has not been shown in any prospective fashion. It has been suggested that because of their relatively good prognosis and non-aggressive course, surgical removal of primary or metastatic cancers may be curative and should be attempted wherever possible [6,15]. Additionally, the combination of interferon (IFN-alpha2a) with retinoids (isotretinoin) seems to be of promise to prevent tumor development in Muir-Torre syndrome [16]. Besides, oral isotretinoin has been reported to prevent the development some of the malignancies in this syndrome. A dosage of as 0.8 mg/kg/d may be effective [3].

In conclusion sebaceous gland tumors are rare and the diagnosis of such a tumor should suggest the possibility of Muir-Torre syndrome and prompt a search for associated malignancies, and for the underlying genetic mutation. Family members should be monitored to detect early cancers and perhaps should be enrolled in chemoprevention trials. Genetic studies can help identify the inherited molecular defect that causes the Muir-Torre syndrome.

## Competing interests

The author(s) declare that they have no competing interests.

## Authors' contributions

**KT**: conceived the study, performed the surgical management and revised the manuscript for scientific content.

**KB**: participated in literature search, study design, and preparation of the manuscript.

**KV**: participated in literature search, study design and in the revision of the study and preparation of its final version.

**TT**: participated in study design, literature search and preparation of the manuscript.

**SA**: participated in study design and literature search.

**DB**: has given the approval for submitting the final version of the manuscript.
